# Community language exposure affects voice onset time patterns in Spanish-English bilingual children and functional English monolingual children

**DOI:** 10.1017/s1366728925000045

**Published:** 2025-02-10

**Authors:** Robert Mayr, Simona Montanari, Jeremy Steffman, Manifa Baghomian

**Affiliations:** 1 Centre for Speech, Hearing and Communication Research, Cardiff Metropolitan University; 2 Department of Child and Family Studies, California State University, Los Angeles; 3 Linguistics and English Language, The University of Edinburgh; 4 Department of English, California State University, Los Angeles

**Keywords:** voice onset time, stop production, functional monolinguals, Spanish-English bilingual children

## Abstract

This study examined English VOT productions by 37 Spanish-English bilingual children and 37 matched functional monolinguals, all aged 3–6 years, from the same Latinx community. It also assessed the bilinguals’ Spanish stop productions and investigated the effects of age and language exposure on their VOT productions. The results revealed credible between-group differences on English voiced, but not voiceless, stops, with shorter VOTs for bilinguals. However, both groups exhibited similar pre-voicing levels, which may suggest an effect of the community language, Spanish, not only on the bilinguals’ English VOT patterns but also the monolinguals’. The study also found cross-linguistic differentiation of voiceless stops, but not voiced ones, in the bilinguals’ productions and revealed effects of age and exposure not only on VOT in Spanish but also in the majority language, English. These findings have important implications for the conceptualization of monolingual-bilingual comparisons in settings where the community and majority language coexist.

## 1. Introduction

To date, the majority of bilingual (BIL) speech development research has focused on children’s production abilities as compared to monolingual (MON) peers without taking into account the sociolinguistic context in which speakers use their language(s). However, in contexts characterized by a high degree of language contact, functional MONs may have some knowledge of the contact language due to indirect exposure and may produce speech and language patterns that, similarly to those of BILs, show features of the contact language (Barlow & Combiths, 2019; Mayr et al., 2017; Mennen et al., 2020). It is thus important to examine the speech patterns of BIL children growing up in high language contact contexts with reference to those of functional MON peers from the same community. In these contexts, BIL speakers typically co-activate their two languages in parallel, which may result in a higher degree of cross-linguistic influence (CLI) – and possibly further differences from MON patterns – than in contexts, which require single language use (Green & Abutalebi, 2013).

This study examines the English and Spanish speech sound productions of BIL Latinx preschoolers growing up in a heavily Spanish-speaking area of Los Angeles. The first goal is to examine the BIL children’s English productions as compared to those of functional MON Latinx peers from the same community. We then examine the extent to which the BIL children have separate categories for their Spanish and English stops and how age and language exposure predict production patterns. In California, Spanish-speaking Latinx represent nearly one-third of the entire population, and 40% of Los Angeles County residents speak Spanish. In the community in which the research was conducted, 83.11% of residents spoke Spanish in 2022 (worldpopulationreview.com). Understanding speech sound development in contexts where BIL speakers constitute a large segment of the population is important not only for clinical and educational purposes but also for theoretical reasons. Indeed, this research can improve the health and educational outcomes of the U.S. Spanish-speaking population while refining theories of BIL speech sound development, incorporating the varying circumstances, in which BILs learn and use their languages.

### 1.1. Bilingual speech sound development

Many studies have examined BIL speech sound development among Spanish-English-speaking children. Research has assessed phonetic inventories, segmental accuracy as measured by overall percentage of correct vowels and consonants, and the acoustic properties of stop consonant production (see Gildersleeve-Neumann & Goldstein, 2022, for a review). Overall, these studies show that, while BIL children may sometimes display production patterns that are similar to those of MONs, BILs’ two languages influence each other in development, a phenomenon known as CLI Fabiano-Smith & Bunta, 2012; Mayr & Siddika, 2018). CLI can lead BILs to experience protracted speech sound development in one or both languages (see, for example, Gildersleeve-Neumann et al., 2008) or produce speech sounds differently from MONs (Fabiano-Smith & Bunta, 2012). At the same time, when one of the BIL’s languages contains a structure that may be difficult or infrequent in the other language, CLI may accelerate the acquisition of that structure compared to MON acquisition, therefore having a facilitative effect on BIL speech sound development (Mayr et al., 2015; Stoehr et al., 2018).

While there is no specific theory to account for speech sound development in young BIL children, Flege’s Speech Learning Model (SLM, Flege, 1995; see also the revised model, the SLM-r, Flege & Bohn, 2021), one of the most influential models of the acquisition of speech in a second language (L2), can explain how children develop speech sounds in two languages in early childhood. The model assumes that L2 speech production and perception co-evolve and proposes that the degree of similarity/dissimilarity between L1 and L2 phones affects L2 speech production. Specifically, the model proposes that the ease with which L2 phones are produced accurately is dependent on the extent to which they map onto L1 categories. When L2 phones are similar to those of the L1, L1 categories act as attractors, and L1 and L2 sounds form merged representations that may have compromise values that differ from those of MONs in both the L1 and L2. In this case, BIL children may acquire only one category for two sounds that they perceive to be alike in the two languages and produce these two sounds as the same. However, when L2 phones are dissimilar to sounds in the L1, they will be perceived and produced more accurately because learners will form new speech sound categories.

As to the effect of the age of the learner, the original SLM (Flege, 1995) predicts that increasing age of acquisition goes hand in hand with a decreasing ability to distinguish L1 and L2 sounds. This hypothesis inversely suggests that an early age of acquisition promotes speech sound discrimination, resulting in more limited CLI and thus more language-specific speech sound production in children than adults (Baker et al., 2008; Tsukada et al., 2004). This is because L1 phonetic categories are less robust/entrenched at a young age, which allows children to more readily recognize novel sound contrasts and form new categories. Hence, young BIL children can be predicted to be less prone to CLI and more likely to acquire and produce native-like sounds in both languages compared with older learners. At the same time, there is increasing evidence that even young BILs commonly exhibit CLI and non-native pronunciations (Casillas & Simonet, 2016; Mayr & Siddika, 2018), which is why age no longer features as a critical factor in the revised SLM (SLM-r, Flege & Bohn, 2021).

While Flege’s original SLM did not ascribe BILs’ differential sound production to differences in language exposure between BILs and MONs, the SLM-r includes the quantity and quality of the L2 input (“language experience”), as variables affecting L2 perception and production. The revised model proposes that “L2 learners gradually ‘discern’ L1-L2 phonetic differences as they gain experience using the L2 in daily life, and [that] the accumulation of detailed phonetic information with increasing exposure to statistically defined input distributions for L2 sounds will lead to the formation of new phonetic categories for certain L2 sounds” (Flege & Bohn, 2021, p. 32). Hence, learners with more L2 experience/input are expected to be more successful at L2 perception and production than learners who have had less experience with the L2.

Several studies have shown that (a) speech sound similarity/dissimilarity, (b) age and (c) exposure affect BIL children’s perception and production of speech sounds. First, sounds that are perceived as similar have been shown to cause more difficulty. For instance, Montanari et al. (2023), who studied English and Spanish perception in BIL 3-to-6-year-olds in the U.S., documented difficulties with the perception of English voiced stops since these are acoustically equivalent to Spanish voiceless consonants. Stoehr et al. (2018), who investigated stop consonant production in BIL children who spoke an aspirating language, German, as the heritage language and a voicing language, Dutch, as the majority language, found similar results, with the BIL children producing Dutch voiced stops, which are pre-voiced, and German voiced stops, which are typically short-lag, with the same, short-lag-like acoustic values. Muru and Lee (2017), who examined Spanish and English stop voicing patterns in 5.5- and 10-year-old BIL children in the U.S., similarly found that only the older children differentiated English and Spanish voiced stops, whereas the younger children displayed the same phonetic categories for these sounds, possibly due to their perceived similarity.

Age has also been shown to predict both perception and production skills in BIL children. As to perception, Montanari et al. (2023) found that age was the only predictor of perceptual performance in the societal language, English, whereas it did not moderate perception abilities in Spanish, the heritage language. The authors argued that as BIL children in the U.S. get older, they interact increasingly more with members of the wider English-speaking community, thereby expanding their opportunities to hear the societal language and improve their perceptual abilities. On the contrary, input in a heritage language typically decreases with age due to English-only schooling, so that older children may not necessarily be the best perceivers of that language. McCarthy et al. (2014) also found that children’s perceptual and productive performance in the societal language, English, improved with age. Indeed, after 19 months of regular and consistent exposure to English in preschool, in an environment where the heritage language was relegated to the home domain, the stop consonant perception and production patterns of their Sylheti/English BIL participants differed from the Sylheti-influenced patterns the children displayed a year earlier and were no longer significantly different from those of MONs. The authors speculated that phonemic categorization and phonetic production may be initially affected by language dominance in young BILs, but these can be acquired and refined with language experience as children increasingly hear and use the weaker language. On the contrary, Stoehr et al. (2018) did not find evidence of age effects in either the societal or the heritage language in their German-Dutch BIL children, possibly because the children were simultaneous BILs who extensively heard both languages from early in life.

Finally, exposure, or the amount of input a child receives in a language, has also been shown to be related to perception and production. In Montanari et al. (2023), exposure solely predicted perceptual performance in the heritage language, Spanish, whereas it did not moderate English perception. Stoehr et al. (2018) similarly found that exposure moderated consonant production in the heritage language, German, whereas it was not related to production abilities in the societal language, Dutch. Similarly to Montanari et al. (2023), Stoehr et al. (2018) speculated that exposure is crucial for the establishment of a language-specific voicing system in the heritage language, which is heard from a smaller segment of society, whereas the impact of exposure is limited for the language that is spoken in the wider society and which children hear across domains. Interestingly, however, in a study of perception and production among preschoolers living in an extensive language contact context – Barcelona, Ramón-Casas et al. (2023) found that exposure was important even for the perception of a language highly spoken in the community. The authors found indeed that Catalan-dominant BILs who had Catalan-speaking mothers reliably outperformed Spanish-dominant BILs who heard more Spanish than Catalan in both the perception and production of the Catalan /e-ε/ contrast. Overall, the results of these studies demonstrate, in line with the SLM-r predictions, that the more input is received in a language, the better the speech perception and production abilities in that language. However, it is unclear whether this holds true for both of BILs’ languages.

### 1.2. The acquisition of the voicing contrast in English and Spanish

The acquisition of the voicing contrast in word-initial stops is a challenging task for BIL children. Like their MON peers, they need to become sensitive to the fine phonetic distinctions in voice onset time (VOT) that signal phonemic contrasts. This is a protracted process that takes several years to complete and involves a number of developmental stages (Macken & Barton, 1980a, 1980b). The task is especially complex for BILs learning languages, in which the voicing distinction is implemented differently, as in the case of Spanish-English BILs.

Three types of stop voicing categories are traditionally distinguished in terms of VOT: (1) pre-voiced stops (or lead voicing), in which voicing occurs before the release of the stop (negative VOT values); (2) short-lag unaspirated stops, in which voicing is simultaneous with the release or occurs shortly thereafter (approximately 1–30 milliseconds) and (3) long-lag aspirated stops, in which voicing occurs with a significant time lag after the release (approximately 30 to 80 milliseconds) (Lisker & Abramson, 1964). Acoustic analyses of stop productions in Spanish and English indicate that Spanish voiced stop consonants are usually pre-voiced; that is, they are characterized by the onset of voicing prior to the release of the stop, whereas the onset of voicing for English voiced stop consonants typically begins shortly after the release of the stop burst, in the short-lag range. On the other hand, Spanish voiceless stops, like English voiced stops, are produced as short-lag and English voiceless stops as long-lag. Therefore, voiceless stops in Spanish overlap with voiced stops in English in VOT values, posing a challenge to BIL Spanish-English speakers. This constellation may lead to CLI and manifest in erroneous phonemic representations and non-target productions.

The acquisition of VOT in English and Spanish has been examined in several MON investigations and in a few BIL small-scale studies. In general, research has shown that the English short-lag versus long-lag voicing contrast is acquired early and is adult-like by two years of age (Macken & Barton, 1980a). On the contrary, the Spanish lead voice versus short-lag voicing contrast is generally acquired late and is not adult-like until at least the elementary school years (Macken & Barton, 1980b; Muru & Lee, 2017). Most researchers have explained the later acquisition of the lead voice/short-lag contrast on the basis of perceptual and articulatory difficulties. Pre-voicing is acoustically less salient (Van Alphen & Smits, 2004) and articulatorily more complex than aspiration (Ohala, 1997). The shorter vocal tracts of children compared with adults, in addition, make sustained voicing even more difficult, providing further aerodynamic challenges. These difficulties may prompt children to make use of compensatory strategies, for example, by realizing word-initial voiced stops as spirants (as shown in Macken & Barton, 1980b, and Mayr & Montanari, 2015).

BIL studies have shown that both developmental factors and CLI affect BIL children’s VOT patterns. For example, in a small study of two typically developing children, Konefal and Fokes (1981) found that while the short-lag range for voiceless Spanish stops had been acquired by age four, pre-voiced Spanish voiced stops did not appear until age seven and were produced as short-lag at earlier ages. However, VOT values for both the voiced and voiceless stop categories were significantly different between Spanish and English by age four, suggesting that, by preschool age, Spanish-English BIL children may have developed separate voicing contrasts for their two languages. Muru and Lee (2017), the largest Spanish-English VOT study involving 32 children, similarly found significant distinctions between English and Spanish stop categories for voiceless stops before voiced ones. Specifically, both the 5-to-6-year-old and the 10-year-old children demonstrated distinctive categories between the two languages for voiceless stops. However, only the older children managed to produce a cross-linguistic contrast for voiced stops, realizing Spanish /b d g/ mostly with lead voicing and English /b d g/ with short-lag VOT values. In contrast, the 5-to-6-year-old used short-lag and pre-voiced realizations indiscriminately in both languages. The authors suggest that the use of pre-voicing in English in the younger children is a result of CLI from Spanish to English. This is in line with findings from adult BILs, which suggest that voiced stops are more likely to be affected by CLI than voiceless ones (e.g., Kang et al., 2016; Schwartz, 2022).

Fabiano-Smith and Bunta (2012) is the only VOT study in Spanish-English BIL children that carried out comparisons with MONs. The study compared the bilabial and velar voiceless stop productions of eight Spanish-English BIL preschoolers to those of eight Spanish and eight English MON peers. The BIL children came from a high language contact immigrant community in Philadelphia, Pennsylvania, where both English and Spanish were widely used in preschool instruction. The children were of Puerto Rican or Dominican descent and spoke Caribbean Spanish. The English MONs came from a different neighborhood in Philadelphia where English was the only language used in school. MON Spanish-speaking children came from south-central Mexico and spoke the Mexican Spanish variety (the authors argued that dialectal variation was not relevant for the sounds under study). The results showed that the BIL children’s VOT productions differed from their MON counterparts in English but not in Spanish, which the authors interpreted as stemming from CLI. Specifically, the children’s VOT values for English /p/ and /k/ were significantly shorter than those of their English MON peers in line with the shorter VOT of Spanish voiceless stops. Although there was a statistically significant difference between the VOT values produced by English and Spanish MONs, the BIL children produced no significant difference in VOT between Spanish and English stops, which was interpreted as a lack of language-based differentiation. Overall, the findings of these studies, although limited in sample size and the sounds that were studied, suggest that (a) Spanish-English BIL children may neither differentiate voiceless nor voiced stops in Spanish and English at preschool age (Fabiano-Smith & Bunta, 2012; Konefal & Fokes, 1981); (b), they may differentiate voiceless but not voiced stops by kindergarten age (Konefal & Fokes, 1981; Muru & Lee, 2017); and (c), they may display different English and Spanish categories for both voiceless and voiced stops by the late elementary school years (Muru & Lee, 2017).

### 1.3. The present study

This study contributes to the literature on Spanish-English BIL speech production by examining the English and Spanish stops produced by 3-to-6-year-old who live in a high language contact community where both Spanish and English are widely used. Our first goal is to compare the BILs’ productions of English /p-b/ and / t-d/ to those of English MON peers from the same community. Since children may take years to produce segments categorically as adults, we compare BIL performance to that of MON peers who are also in the process of refining their categories. However, this study is the first to compare BILs’ productions to those of functional MON peers who may have some knowledge of the contact language due to indirect exposure and may produce speech and language patterns that, similarly to those of BIL speakers, show features of the contact language (Mayr et al., 2017; Mennen et al., 2020). Indeed, it has been shown that MONs who live in language contact situations may have some phonetic knowledge of the community language. For example, in Barlow and Combiths (2019), American English MONs from Southern California who reported no knowledge of Spanish produced laterals and stops differently across Spanish and English in a BIL task. We limit the BIL-MON comparisons to English because the participants are growing up in the U.S., and we feel it is inappropriate to compare the participants’ Spanish production skills to those of Spanish MON peers living in a Spanish-speaking country, as Spanish input in a heritage language setting will undoubtedly be less rich than in a societal language context with consequences on speech sound development (Bayram et al., 2021).

We then focus on the extent to which the BIL children have separate categories for their Spanish and English stops. As reviewed above, both English and Spanish contrast voiced and voiceless categories, but the phonetic realizations of the two categories differ between the two languages as assessed through VOT, and young BILs may fail to differentiate Spanish and English stops at preschool/early school age (Fabiano-Smith & Bunta, 2012). We specifically focus on stop production at two places of articulation, bilabial and coronal – thus excluding velars – in order to strike an optimal balance between examining place of articulation effects and making our production task feasible for young children, similarly to other production studies of young populations (Fabiano-Smith & Bunta, 2012; McCarthy et al., 2014; Ramón-Casas et al., 2023).

Finally, since age and exposure have been shown to affect BIL speech sound perception (McCarthy et al., 2014; Montanari et al., 2023; Ramón-Casas et al., 2023) and production (McCarthy et al., 2014; Ramón-Casas et al., 2023; Stoehr et al., 2018), we examine the extent to which both variables predict English and Spanish production patterns. The study thus addresses the following research questions (RQs):

(**RQ1**) Do Spanish-English BIL children living in a context of high language contact produce English stop categories differently from functional English MONs from the same community?

(**RQ2**) Do the BILs have separate categories for their Spanish and English stops?

(**RQ3**) Do age and exposure predict the BIL children’s English and Spanish stop production patterns?

As for RQ1, Fabiano-Smith and Bunta (2012) found BIL-MON differences. However, the study only included eight BIL children, was limited to voiceless stops, and involved MONs from a community that was different from that of the BILs. Since our study compares BIL children’s productions to those of functional English MON peers from the same BIL community, it is possible that the latter have some Spanish knowledge due to indirect exposure, and they possibly produce Spanish-influenced speech patterns similarly to the BILs. We are therefore unsure whether our results will match the BIL-MON differences found in Fabiano-Smith and Bunta (2012).

As for RQ2, and relatedly RQ3, we hypothesize that our participants will have separate categories for Spanish and English voiceless stops while failing to show differentiation for voiced stops since target-like pre-voicing, as required in Spanish, has been shown to be acquired late. However, older children and children with more exposure to Spanish are expected to produce increased pre-voicing and larger levels of differentiation of English and Spanish stops.

## 2. Method

### 2.1. Participants

A total of 74 (3-to-6-year-old) children participated in the study, 37 (24F, 13 M) of whom were Spanish-English BILs) and the remaining 37 (23F, 14 M) were classed as functional English MONs. The children were recruited from the same BIL community in Southern California where Spanish and English are used as community languages and assessed by trained experimenters proficient in Spanish and English. Only children without a documented history of speech, language, hearing, cognitive or neurological deficits according to parental reports were included in the study. At the time of the study, the BIL children had a mean age of 51.16 months (SD = 9.55), which did not differ from that of the MON children, which was 48.95 months on average (SD = 7.41, *t* (73) = 1.099, *p* = .138). Moreover, the BIL children did not differ from the MON peers in terms of the number of males and females included (χ^2^ = .058, *p* = .809), and the two sets of children were matched in terms of socioeconomic status as they all came from low-to-middle income families from the same community. The BIL children were all Mexican-heritage, simultaneous BILs raised in homes in which they had regular daily exposure to both Spanish and English through family members from an early age. All BIL children spoke Spanish and English fluently, as assessed informally by the Spanish-English BIL experimenters, although they varied in their language exposure and use (see exposure patterns below).

The MON children were members of the same Latinx community in Southern California as the BIL children; they were also of Mexican heritage and hence exposed to Spanish to some extent in the wider community. However, they mainly heard English from family members in their homes and, in line with Best and Tyler’s (2007), p.16) definition of functional MONs, they were “not actively learning [Spanish] and […] linguistically naïve to [it].” Accordingly, since they had no knowledge of Spanish and did not use the language actively based on parental reports, they were classed as functional MONs rather than receptive or incipient BILs (Diebold, 1964). Note, however, that the children’s lack of knowledge in Spanish was not formally assessed, and that based on previous work (Barlow & Combiths, 2019), it cannot be ruled out altogether that the functional MONs had some rudimentary knowledge of Spanish.

The information on the children’s background was provided by caregivers in a detailed questionnaire. This also included information on children’s exposure levels to Spanish and English. Exposure was measured on a Likert scale from 1 to 5, with 1 representing “child hears mostly Spanish,” 2 “child hears more Spanish than English,” 3 “child hears as much Spanish as English,” 4 “child hears more English than Spanish” and 5 “child hears mostly English.” While a range of alternative measures for exposure would have been available, such as an assessment of absolute exposure levels in terms of the number of hours children hear Spanish and English, we adopted our relative measure of language exposure since it was time-efficient, parent-friendly, and has been shown to estimate a child’s overall BIL experience reliably (Calandruccio et al., 2021). On that basis, the BIL children’s average exposure score was 2.86 (SD = 0.87, range: 1–4), while that of the MON children was 4.7 (SD = 0.46, range: 4–5), a difference that was found to be statistically significant (*t*(73) = 11.171, *p* < .001). This confirms that the MON children hear more English and less Spanish than the BIL children.

### 2.2. Experimental materials

To elicit productions of voiced and voiceless bilabial and coronal stops in Spanish and English, a child-friendly picture naming task was conducted in which the children were recorded naming pictures displayed on a computer screen. The target materials encompassed 3 (words) × 4 (stops) = 12 words in each language, with each word starting with a singleton bilabial or coronal stop in its onset (see Table 1). These were carefully selected following piloting on a larger set of items so as to strike an optimal balance between children’s word familiarity, imageability and control of phonetic context effects. With respect to the latter, we matched the words prosodically across the languages as much as possible, ensuring that all items were bisyllabic with a trochaic pattern and that the target stops were followed by an open vowel. All items were nouns, and only high-frequency words that young children were expected to be familiar with were included.

### 2.3. Procedure

The data were collected as part of a larger project on speech perception and production in Spanish-English BIL children, although this study only reports on production. Data collection was conducted in individual sessions on the premises of a university research laboratory. For the production task, the children were seated at a comfortable distance from a computer and were asked to name the items displayed on the screen. In the case of no response, children were given prompts or were allowed to repeat the answer provided by the examiner, as in previous studies (Munro et al., 2005). The BILs were asked to name target words in Spanish and English, while the MONs only named English words. To set the BILs in a MON language mode (Grosjean, 2001), to the extent that this is possible in a BIL experiment, data collection of the Spanish and English items was maximally spaced out within a single data collection session on the same day, with a change of experimenter (from an English-speaking to a Spanish-speaking one and vice versa) and a time set for play, conversation and verbal instructions in the target language before beginning the task. Half of the children completed the production task in English first and then in Spanish, while the other half completed it in Spanish first and then in English. Recordings were made using a TASCAM DR-07X stereo handheld digital audio recorder, which was positioned a few centimeters from the participant’s mouth.

### 2.4. Analysis

The children’s VOT patterns were analyzed acoustically using PRAAT software (Boersma & Weenink, 2021). Measurements were taken from the release burst of each item, as represented by a sharp peak in waveform energy, to the onset of voicing of the following vowel, as signaled by the zero crossing of the first glottal pulse (Supplementary Figure S1 (a), S1(b)). Where more than one transient was visible, measurements were taken from the first release burst. If voicing commenced before the release, that is, during the closure phase of the stop, VOT was measured from the point when vocal fold vibration was visible in the waveform, alongside aperiodic wide-band energy in the spectrograms, up to the release burst (Supplementary Figure S1(c)). Tokens where this could not be established clearly were discarded. In total, the children produced 12 (words) × 74 (children) = 888 words in English and 12 (words) × 37 (children) = 444 in Spanish, for a total dataset of 1,332 words. Of these, 50 English items (5.6%) and 31 Spanish items (6.98%) had to be discarded due to poor recording quality or inaccurate productions, leaving 838 English tokens and 413 Spanish tokens for analysis.

### 2.5. Statistical analysis

To address our research questions, we used Bayesian linear mixed effects modeling, implemented using *brms* (Bürkner, 2017, 2018) in the RStudio Environment (R Core Team, 2016). Model summary tables are included in the Supplementary Materials of the paper (Supplementary Tables S1 to S4). All data, analysis code, Supplementary Details on model specifications, and saved model objects are also hosted as open access on OSF at: https://osf.io/pcz5f/

We considered two dependent variables. The first was VOT, as measured in milliseconds. These values could be negative (pre-voicing), zero or positive (short-lag and long-lag aspirated stops). The values were submitted to a cube-root transform, explained in the Supplementary Materials. The second dependent variable in the analyses was a binary representation of the presence or absence of pre-voicing, applied only to the stops /b/ and /d/ (dropping /p/ and /t/ observations from the analysis). The added benefit of this analysis is that it allows us to consider voicing as binary, abstracting over actual VOT values, and providing possible additional insights into the data.

The results are organized in two parts in line with our research questions. In the first part, we present the results from a comparison of the BIL and MON children’s stop productions; in the second part, we compare the BIL children’s stop productions across languages. In this latter analysis, we examine the effects of age and exposure on BIL children’s stop productions in Spanish and English. In the first analysis, we predicted the dependent variables as a function of *group* (contrast coded in the model representation with −0.5 mapped to BIL and 0.5 mapped to MON), *place of articulation* (bilabial mapped to −0.5, coronal mapped to 0.5) and *voicing category* (voiced mapped to −0.5 and voiceless mapped to 0.5). Note here that voicing refers to the phonological voicing category: /b/ and /d/ are designated as voiced and /p/ and /t/ as voiceless. The random effects in the model included random intercepts for both *participant* (speaker) and *item*, where item refers to the word that was produced. There were random by-participant slopes included for all within-participant fixed effects (that is, place, voicing and their interaction). Note that, in the analysis, which considered binary voicing in /b/ and /d/ only, the variable voicing was absent from the model formula.

The second set of analyses was concerned with mediating factors in BILs’ productions of both English and Spanish. Only the BIL speakers were analyzed, predicting the dependent variable as a function of *language* (English mapped to −0.5, Spanish mapped to 0.5), *place* and *voicing*. Two additional variables were entered into the analysis: *participant age* and *exposure*. Both of these numerical variables were scaled and centered. The random effects in the model were again random intercepts for *participant* and *item* and by-participant random slopes for all within-*participant* fixed effects. Both models were fit with weakly informative priors.

Finally, it is important to note how we make inferences from the models. To evaluate the evidence for effects, we make use of common practices for inference from Bayesian model estimates. We report the median estimate for a given fixed effect as well as 95% credible intervals (CrIs). These intervals indicate the range in which 95% of the estimated effect falls. To evaluate if the effect is robust, we consider whether or not these 95% CrIs include or exclude the value of 0. Specifically, when 95% CrI for an effect *includes* the value of zero, this indicates that a non-trivial portion of the estimates are at or very near zero; that is, there is no estimated effect. Moreover, it indicates substantial variation in the estimated *directionality* of an effect: whether the effect is positive or negative. On the other hand, 95% CrI, which *exclude* the value of zero, indicate that the effect is likely not to be zero and further that the directionality is highly consistent. We additionally report another metric, which characterizes the strength of evidence for an effect and, like CrI, represents the disposition of a particular posterior distribution: “probability of direction,” abbreviated pd (Makowski et al., 2019a, 2019b). A distribution centered precisely on the value of zero, that is, no evidence for an effect, would have a pd of 50, that is, 50% of the estimates are positive and 50% are negative. A consistently estimated positive or negative effect, which totally excludes 0, on the other hand, will have a pd of 100. When 95% CrI exclude the value of zero, pd is equal to 97.5, which we take as a (somewhat arbitrary) threshold for indexing an effect as reliable and robust. Values of pd approaching this threshold can also be taken to provide graded evidence for an effect, i.e., be directly interpreted as the models’ estimate of the probability that an effect exists with a particular directionality, with the posterior as a continuous distribution of estimated effects. In adopting the aforementioned threshold, we also note this can be mapped roughly to a frequentist p-value of <0.05 (Makowski et al., 2019b).

We also computed marginal contrasts of interest using *emmeans* (Lenth, 2023) in order to examine interactions, which are present in the model summaries. As with the other estimates, we present 95% CrI and pd, which, when applied to a marginal mean, indicate the models’ estimated value for that combination of effects, and, when applied to a marginal *contrast*, indicate the estimated difference between two or more conditions: in this latter case, 95% CrI, which exclude 0 indicate robust evidence for a non-zero difference between the contrasted conditions.

## 3. Results

### 3.1. Bilingual children’s production of English stops as compared to monolinguals

**Figure 1.**
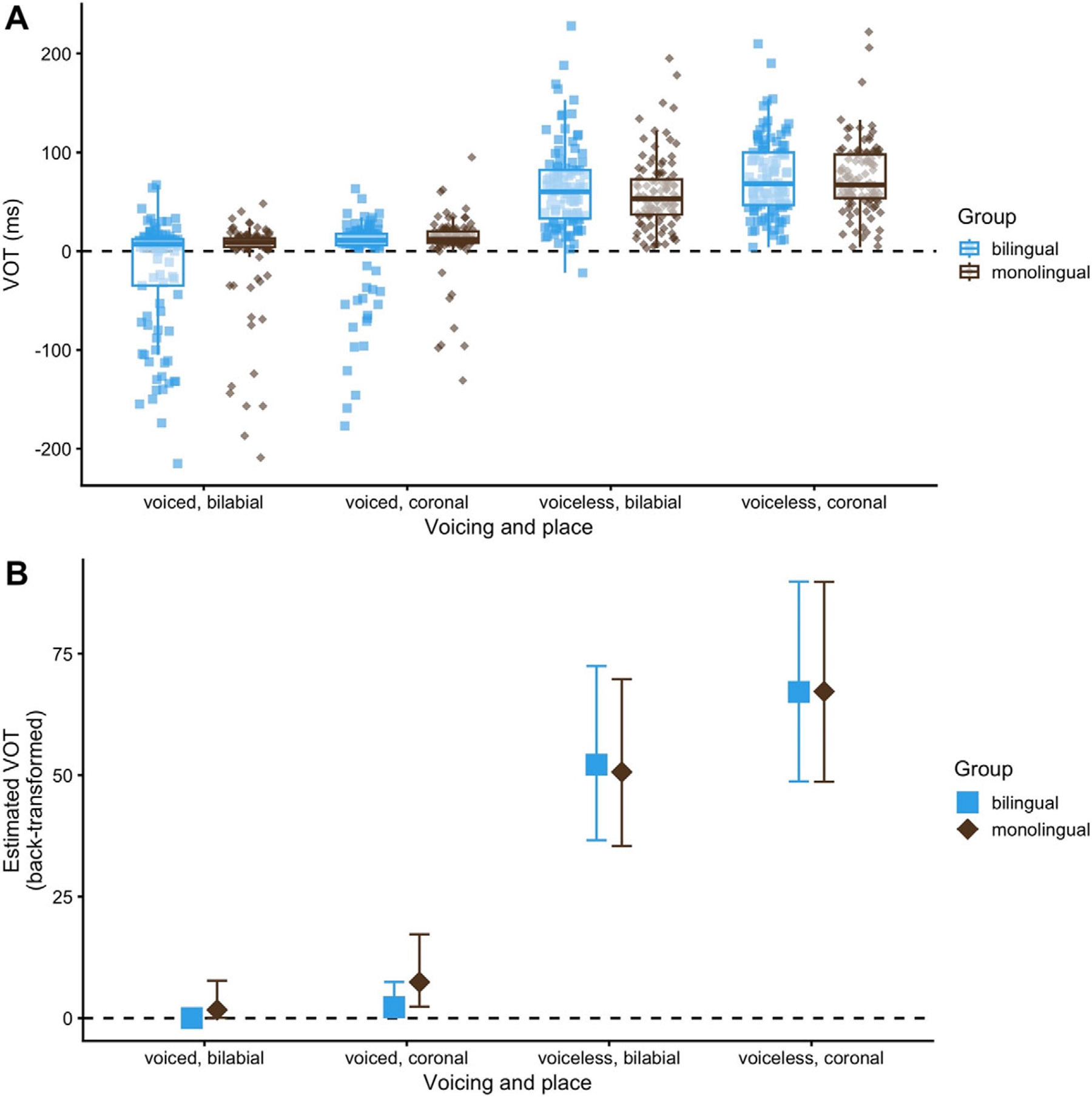
Panel A: raw data points and boxplots for bilingual and monolingual productions of English stops, split by place of articulation and voicing. Panel B: model estimates (median and 95% CrI), split by place of articulation and voicing.

In our first analysis, we were interested in the difference between MON and BIL speakers. Table 2 (left) depicts the mean VOT (in ms) alongside standard deviations and ranges of the English stops produced by the two sets of children. The model estimates lead us to conclude that there was a robust effect of both *place of articulation* and *voicing* on VOT values. Both coronal *place* of articulation ($\hat{\beta }$ = 0.65, 95CrI = [0.24, 1.05], pd = 100) and voiceless voicing category ($\hat{\beta }$ = 2.74, 95CrI = [2.24,3.24], pd = 100) increased VOT values, in line with our general expectation based on place-dependent and voicing-dependent influences on VOT (note that estimates are in the cube-rooted values passed to the model). Figure 1 panel A and B shows these effects, panel A representing the raw data and panel B representing model estimates and 95% CrI. Notably, there was no robust evidence for an interaction between *place* and *voicing* (pd = 94), though the weaker evidence for an interaction likely stems from the fact that the estimated place effect is larger for voiced ($\hat{\beta }$= −0.94) as compared to voiceless ($\hat{\beta }$ = −0.35) stops. In the absence of robust evidence for credible two-way interaction, we, however, conclude the effects of voicing and place of articulation are largely additive.

There was no robust evidence for a main effect of *group*, though there was weaker evidence for an effect (pd = 96), and there was robust evidence for a two-way interaction between *group* and *voicing* ($\hat{\beta }$ = −0.85, 95CrI = [−1.67, −0.03], pd = 98). To inspect the interaction further, marginal contrasts for the group-level difference within each voicing category were computed using *emmeans*. Reflecting the interaction, it was observed that there was credible evidence for a difference between BILs and MONs in voiced stop VOT ($\hat{\beta }$ = −0.83, 95CrI = [−1.62, −0.00], pd = 98) but not in voiceless stop VOT ($\hat{\beta }$ = 0.02, 95CrI = [−0.29, 0.32], pd = 54). On this basis, we can conclude that BILs produced more negative and shorter positive VOT values for /b/ and /d/ than MONs, which can be seen in both panels of Figure 1. Conversely, the groups are quite similar for /p/ and /t/. This further suggests that the weaker main effect of group is driven by voiced stops specifically.

We now turn to the model which analyzed binary pre-voicing in /b/ and /d/ only. The intercept of the model, which, given the coding scheme for the variables, represents the overall estimate of the log-odds of pre-voicing, was negative, and the 95CrI excluded the value of zero in log-odds, where zero is equivalent to a 50% chance of pre-voicing ($\hat{\beta }$ = −2.62, 95CrI = [−3.61, −1.67], pd = 100). This confirms what can be seen in panel A of Figure 1: that a minority of English /b/ and /d/ productions are pre-voiced. Beyond this, the model found no credible evidence for an effect of *place of articulation* (pd = 91) nor *group* (pd = 94), though in both cases, the skew of the distributions leans toward bilabial stops increasing the probability of pre-voicing and BILs being more likely to pre-voice. In the raw data, 16 out of the 37 BILs (that is, 43%) produced pre-voiced items (53/219 tokens in total), and 13 out of the 37 MONs (that is, 35%), for a total of 25/209 tokens.

### 3.2. Bilingual children’s stop productions in English as compared to Spanish

**Figure 2.**
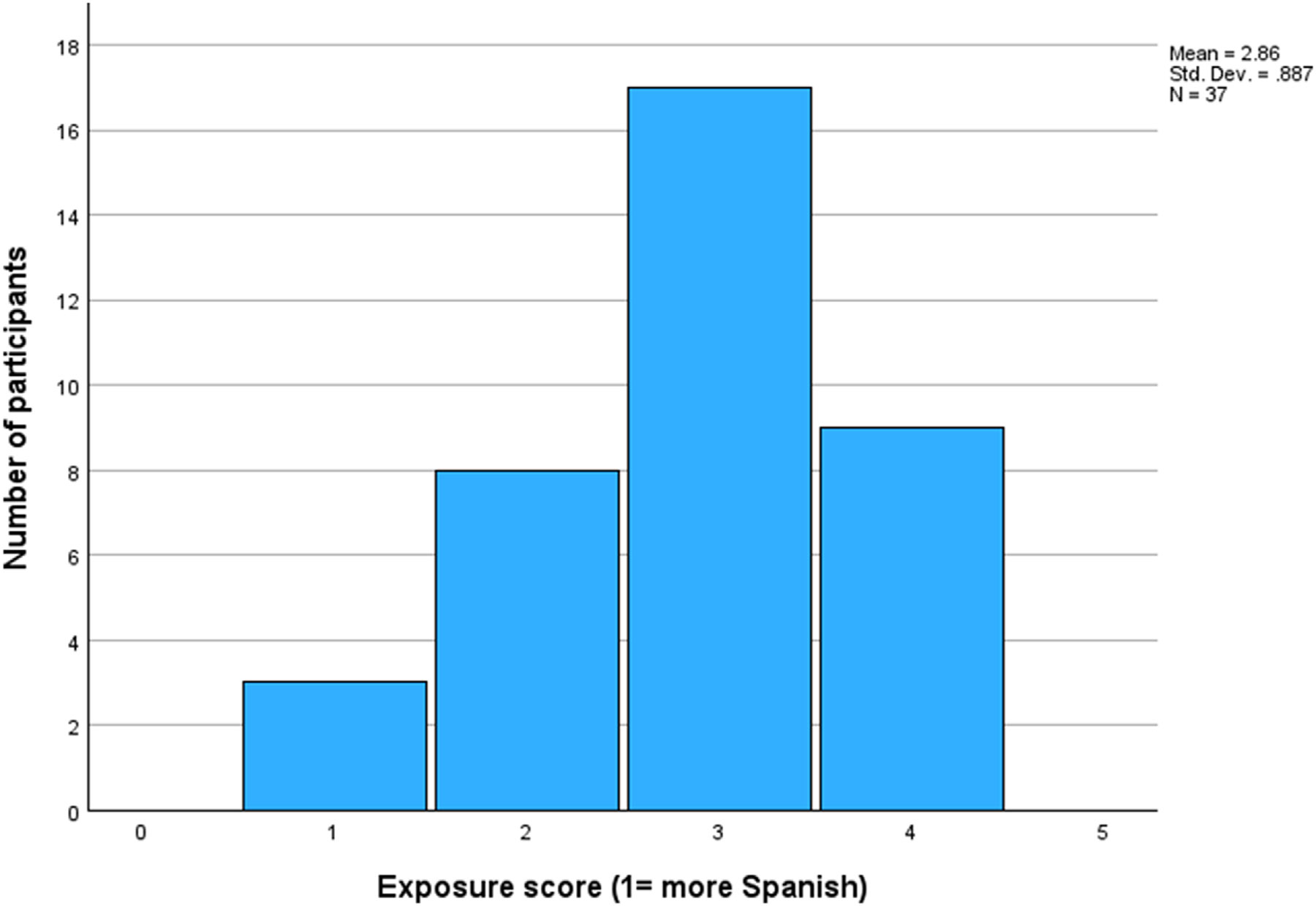
Histogram of bilingual children’s exposure score.

In our second analysis, we evaluated how the same BIL children produced both English and Spanish stops while crucially considering their age and exposure as predictors. Recall that children’s exposure to Spanish versus English was examined using a 5-point Likert scale, ranging from 1 “child mostly hears Spanish” to 5 “child mostly hears English.” The histogram in Figure 2 shows that the BILs varied in their exposure levels, with the majority having a score of 3, “child hears as much Spanish as English.” Since only 3 children had a score of 1 (“child mostly hears Spanish”), for the purposes of the following analyses their scores were combined with those having a score of 2 (“child hears more Spanish than English”).

**Figure 3.**
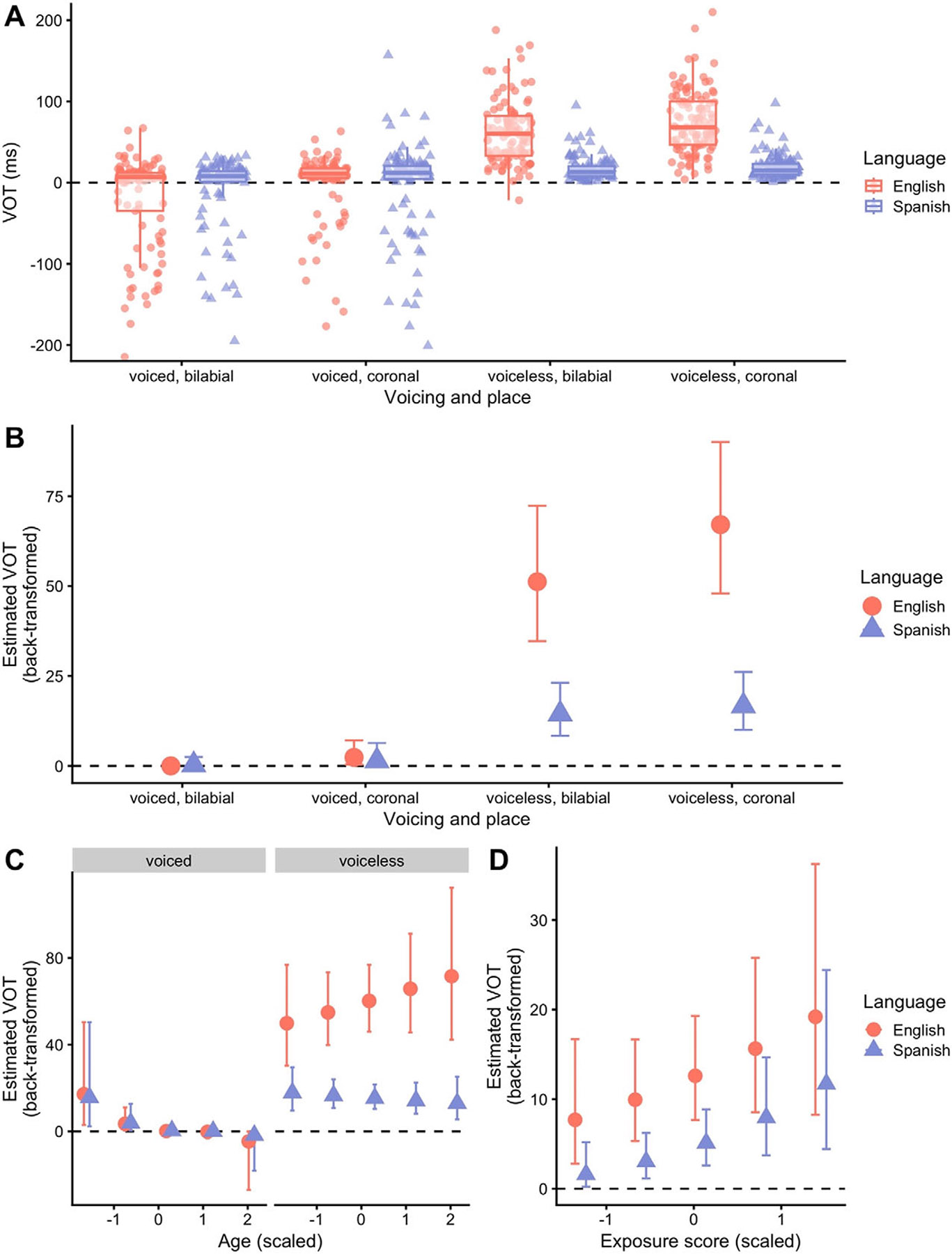
Panel A: raw data points and boxplots for bilingual productions of English and Spanish stops, split by place of articulation and voicing. In panel A, one observation is not shown because it had a negative VOT under – 200 ms, which we used as the minimum for the y-axis. Panel B: model estimates (median and 95% CrI), split by place of articulation and voicing. Panel C: model estimates as a function of age and language. Panel D: model estimates as a function of exposure score and language.

Before considering the influence of exposure and age, we will present the findings for the other variables in the model, which are shown as raw data and model estimates in Figure 3 panels A and B, respectively. Table 2 (right) also depicts the mean VOT values (in ms) with standard deviations and ranges of the BIL children’s Spanish stops. The model finds a credible main effect of *place* ($\hat{\beta }$ = 0.54, 95CrI = [0.22, 0.87], pd = 100), showing an estimated increased VOT for coronal stops. A main effect of *voicing* is also clear and large in magnitude, showing that, as expected, voiceless stops have higher VOT values ($\hat{\beta }$ = 2.39, 95CrI = [1.88, 2.91], pd = 100). There was also notably a main effect of *language*, showing that Spanish productions have estimated lower VOT ($\hat{\beta }$ = −0.62, 95CrI = [−0.98, −0.25], pd = 100). Each of these effects is shown in panel A (raw data) and panel B (model estimates) in Figure 3. One notable pattern that is also apparent in these figures is the presence of a credible interaction between *language* and *voicing* ($\hat{\beta }$ = −1.55, 95CrI = [−2.37, −0.72], pd = 100). To inspect this interaction, the language difference between each voicing category was computed using *emmeans*, showing that, as is evident in the figures, there was not a reliable difference across the languages for voiced stops ($\hat{\beta }$ = −0.16, 95CrI = [−0.82, 0.49], pd = 69), whereas there was one for voiceless stops ($\hat{\beta }$ = 1.39, 95CrI = [0.97, 1.82], pd = 100), with Spanish productions having shorter VOT in /p/ and /t/ ($\hat{\beta }$ = 2.49) than English productions ($\hat{\beta }$ = 3.88).

Next, we turn to the effect of (scaled) age and exposure. Age showed a credible main effect on estimated VOT values ($\hat{\beta }$ = −0.52, 95CrI = [−0.82, −0.22], pd = 100), and critically interacted with *voicing* ($\hat{\beta }$ = 1.11, 95CrI = [0.60, 1.61], pd = 100). To examine these effects, panel C of Figure 3 plots the *age* effect for each voicing category. Importantly, there was no evidence for a three-way interaction with *language* (pd = 81); however, we present these estimates for each language so that it is clear to the reader how each language patterns. To quantify the age effect in the interaction, we used the *estimate_slopes* function from the *modelbased* package (Makowski et al., 2020). The interaction reflects the fact that there is a robust effect of age on the VOT of voiced stops ($\hat{\beta }$ = −1.08, 95CrI = [−1.59, −0.56], pd = 100), whereby increases in age are associated with decreases in VOT. Conversely, no evidence for an effect is present in voiceless stops ($\hat{\beta }$ = 0.03, 95CrI = [−0.17, 0.22], pd = 61). In the language-specific estimates for the age and voicing effect, it can be noted that the effect is credible for voiced stops in both languages and is of a similar magnitude ($\hat{\beta }$ = −1.15 in English and $\hat{\beta }$ = −1.01 in Spanish). Conversely, while there is a positive trend for English voiceless stops ($\hat{\beta }$ = 0.13) and a negative trend for Spanish stops ($\hat{\beta }$ = −0.07), neither of these effects were credible (pd = 81 and 72, respectively).

Finally, we turn to the effect of *exposure* in the model, which showed a credible positive estimate ($\hat{\beta }$ = 0.33, 95CrI = [0.02, 0.63], pd = 98) visualized in panel D of Figure 3. Here, too, there was not a credible interaction with language, but we present the estimates for each language separately. As is shown in the figure, numerical increases in exposure, equivalent to *more English exposure*, result in increases in VOT. As is clear from the estimates, the effect is fairly analogous in each language (i.e., no two-way interaction).

**Figure 4.**
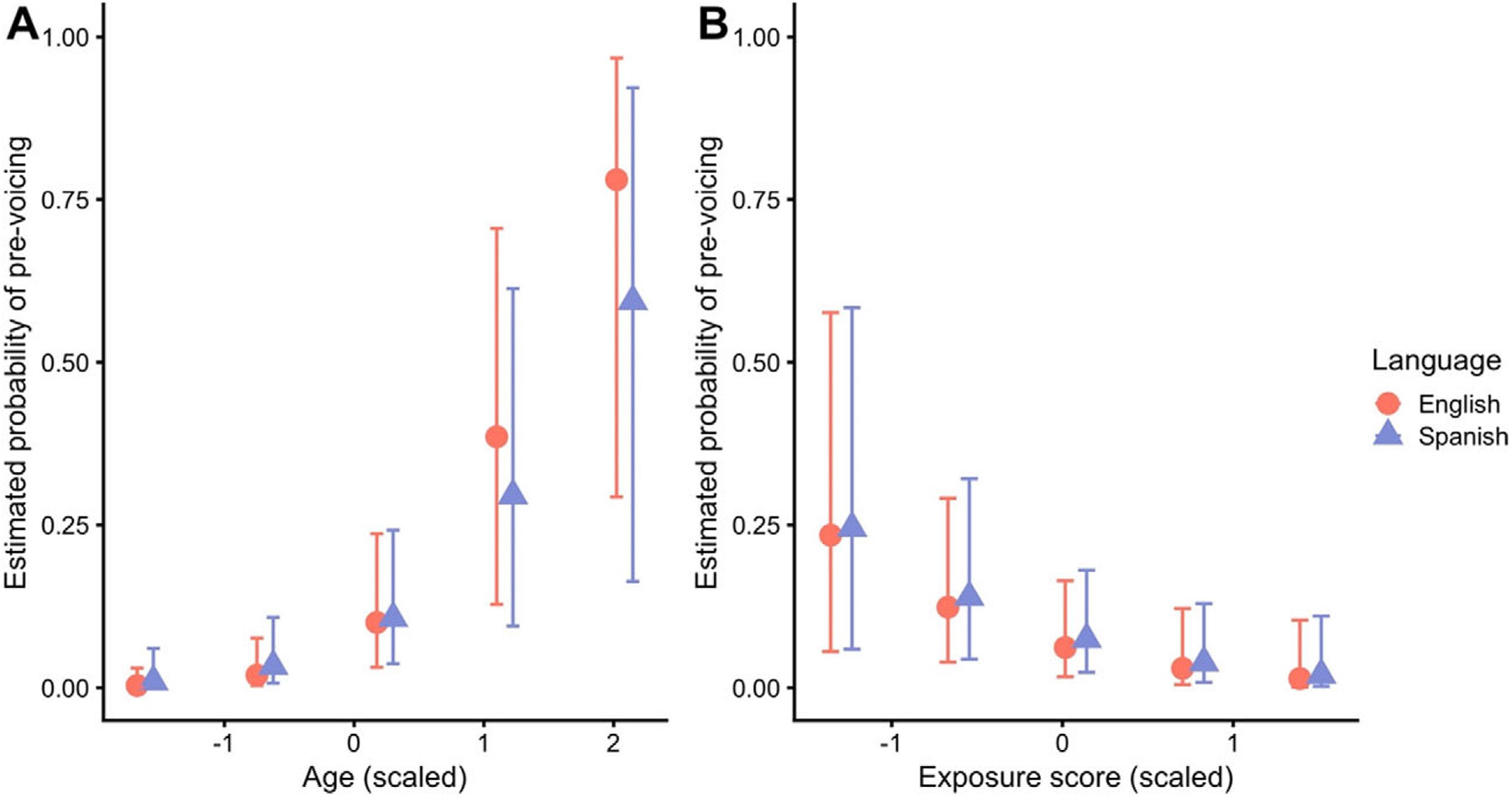
Panel A: model estimates from the logistic regression analysis estimating the probability of a pre-voicing response as a function of age, split by language. Panel B: model Estimates as a function of exposure score, split by language.

The model that evaluated binary pre-voicing outcomes (for /b/ and /d/ only) largely comported with these results. There were only two credible effects in the model, both of which are visualized in Figure 4. As shown in panel A, increases in *age* predict an increased probability of pre-voicing ($\hat{\beta }$ = 1.63, 95CrI = [0.82, 2.54], pd = 100). This effect did not interact with any other variable, but both languages are shown to visualize the estimates for each. It can be noted that the pattern is similar across both languages. The effect of exposure is shown in panel B. Also mirroring the continuous VOT model, *exposure* showed a credible main effect ($\hat{\beta }$ = −1.07, 95CrI = [−2.00, −0.18], pd = 99), which did not interact with any other effects, wherein numerical increases in exposure (equivalent to more English exposure) predicted a decrease in the probability of pre-voicing.

## 4. Discussion

This study aimed to advance our understanding of stop consonant production in BIL children by addressing three principal research objectives. First, we examined the extent to which Spanish-English BIL children, aged 3–6 years, differed in their VOT patterns of English bilabial and coronal stops from those of age-, gender- and SES-matched functional English MONs from the same BIL community. Second, the study aimed to determine whether the BIL children produce distinct stop voicing categories for bilabial and coronal stops in Spanish and English. And finally, we investigated the extent to which the BIL children’s stop productions in the two languages are mediated by their age and exposure to the two languages. To the best of our knowledge, this study is the first to compare BIL children’s speech patterns to those of functional MONs from the same community. As such, it incorporates into its design a critical factor that is disregarded in studies involving comparisons with MON children in (often geographically distinct) MON settings: a match in the children’s sociolinguistic reality, including regular exposure to the community language, which, in turn, has significant cognitive consequences in terms of language activation (Green & Abutalebi, 2013). The study is also only the second to compare VOT productions in Spanish-English BIL children with those of MONs but contributes a much larger sample than the only other existing one, that is, Fabiano-Smith and Bunta (2012).

Our first analysis revealed credible effects of *place of articulation* and *voicing* on English VOT patterns, with lower VOT values in bilabials than coronals and in voiced stops than voiceless stops. The effect of place of articulation is expected due to differences in the cavity sizes behind the articulators in bilabials versus coronals, which result in differences in air pressure (Cho & Ladefoged, 1999). The effect of voicing, in turn, suggests that the two sets of children have developed separate categories for voiced and voiceless stops in English. Moreover, the analysis revealed no evidence of a credible difference in VOT between the Spanish-English BILs and the functional English MONs on voiceless stops. Both produced English /p/ and /t/ largely within the long-lag VOT range in line with adult targets and hence provided evidence for successful acquisition of aspiration. Previous work has indeed shown early acquisition of English aspiration patterns by MON children (Macken & Barton, 1980a), BIL children (Kehoe et al., 2004) and trilingual children (Mayr & Montanari, 2015), although in some instances, BILs evidenced overly long VOT values so as to keep voiceless categories maximally distinct across languages (Fabiano-Smith & Bunta, 2012; Mack, 1990; Simon, 2010). The subject in Mack (1990), for instance, produced non-target-like, aspirated realizations for French voiceless stops (mean VOT: 66 ms) but differentiated those from English ones (mean VOT: 108 ms). In the present study, there were a few individual instances of MON and BIL children overshooting the target for /p/ and /t/, but the majority were in line with adult-like patterns.

On voiced English stops, in contrast, the two sets of children exhibited credible differences in VOT, with BILs producing /b/ and /d/ with lower VOT values than MONs. Moreover, our binary analysis, in which items were classed in terms of the presence or absence of pre-voicing, showed that, in line with much of the literature on English voiced stops, both groups realized the two voiced stops mainly within the short-lag VOT range. At the same time, the BILs and MONs also produced a considerable number of pre-voiced items (BILs: 32.38% on /b/; 17.76% on /d/; MONs: 16.04% on /b/; 7.77% on /d/). These items were fairly dispersed across speakers, with 16/37 BIL children (that is, 43.24%) producing pre-voiced tokens, and 13/37 MON children (that is, 35.13%). However, despite the higher percentage in the former, evidence for a group difference in pre-voicing patterns turned out to be weak in our binary analysis. This suggests that while the BILs exhibited lower VOT values for English voiced stops than the MONs, this was not mainly driven by differences in the *number* of pre-voiced items but by differences in the *values* of pre-voiced and short-lag VOT items.

Nevertheless, the pre-voicing patterns observed here require further consideration. After all, the BILs’ patterns for /b/ and /d and the MONs’ for /b/ are substantially higher than in some of the previous literature on English voiced stops. For example, pre-voicing only occurred in around 2% of instances in speakers from Boston (Miller et al., 1986; Volaitis & Miller, 1992), as well as Tennessee and Florida (Morris et al., 2008), in approximately 3% of speakers from Wisconsin (McMurray et al., 2013), and in approximately 7% of speakers of Standard Southern British English (Docherty, 1992). Note that these studies all report data from adult populations. This matters since lead voicing is generally acquired late by MON and BIL children due to its lack of acoustic salience (Van Alphen & Smits, 2004) and articulatory complexity (Ohala, 1997). For instance, in Gandour et al. (1986), MON Thai-speaking children only mastered it at age 7, and in Khattab (2000), MON Arab-speaking children only at age 10. The fact that the 3-to-6-year-old BIL children in the present study exhibited considerable pre-voicing rates in English, despite the perceptual and articulatory complexities associated with it, suggests that their productions may have been influenced by Spanish via CLI. This interpretation is consistent with that put forward in Stoehr et al.’s (2018) study of German-Dutch BIL preschoolers in the Netherlands, which reported pre-voicing in 30% of German /b/ tokens and 21% of German /d/ tokens, although, like English, German voiced stops are typically realized with short-lag VOT values. It also aligns with much of the literature on adult BILs, which found CLI to occur more commonly in voiced than voiceless stops (Kang et al., 2016; Schwartz, 2022).

As we saw, the functional MONs in the present study also produced a considerable number of voiced stops with a voicing lead, in particular for English /b/. Accordingly, their voiced stop realizations may have also been influenced by exposure to Spanish. Recall that these children, whilst unable to use Spanish productively, overhear the language regularly in their BIL community and even from some family members. In previous studies, it has been shown that this kind of experience may have profound consequences. For example, Au and their associates (Au et al., 2002, 2008; Knightly et al., 2003) showed that individuals who had overheard Spanish as children outperformed L2 learners without childhood experience in an adult language class based on acoustic analyses and ratings by native Spanish speakers. Regular overhearing of Spanish in the community may hence have affected the functional MONs’ English voiced stop categories in the present study. This would be consistent with exemplar theoretic approaches to speech processing (Pierrehumbert, 2001, 2003), according to which cognitive representations of speech are very detailed, and individuals implicitly learn phonetic distributions of segments and words. Regular exposure to pre-voiced tokens of /b/ and /d/, even if in another language, could hence have affected the children’s exemplar cloud. In the present study, it is also possible that the children heard such tokens from Spanish speakers, whose productions are likely to be Spanish-accented (Flege & Eefting, 1987), and/or from speakers of Chicano English, a contact variety used by Mexican Americans in which voiced stops are commonly realized with lead voicing or replaced by spirants (Fought, 2003; Santa Ana & Bayley, 2008).

However, while CLI and Spanish-accented English may have affected the MON and BIL children’s English voiced stop productions, one needs to be cautious with this interpretation. After all, the accent of the children’s input providers was not formally assessed in the present study. Moreover, we do not have any data on the pre-voicing patterns of MON English speakers from Southern California. While some studies on other varieties of English have shown low pre-voicing rates (Docherty, 1992; Miller et al., 1986; Morris et al., 2008; Volaitis & Miller, 1992), other studies, in particular, those on southern American English varieties in the US, have shown high pre-voicing rates in MON English adults (e.g., Alabama: 70% for /d/ (Flege & Eefting, 1986); 63% for /b d g/ (Flege & Eefting, 1987); Alabama & Mississippi: 78% for /b d g/ (Hunnicutt & Morris, 2016); Mississippi: 62% for /b d g/ (Herd, 2020)). Additional data are hence necessary to elucidate English pre-voicing patterns, in particular, data comparing their occurrence in MON speakers living within and outside language contact situations.

Our second analysis examined the BILs’ stop realizations in Spanish and English. The results revealed credible main effects of *place* and *voicing*, indicating that, as expected, coronal and voiceless stops, respectively, were produced with higher VOT values. Moreover, more importantly, they also exhibited a credible main effect of *language* as well as a credible interaction between *voicing* and *language*. Follow-up analyses using *emmeans*, in turn, indicated that the BILs only produced a clear cross-linguistic contrast across their voiceless categories, with Spanish /p/ and /t/ realized with short-lag VOT values and English /p/ and /t/ with long-lag ones. In contrast, their productions of /b/ and /d/ did not differ across the two languages, suggesting that they only have a single cross-linguistically merged category for /b/ and /d/. These findings are in line with those of Muru and Lee (2017), who found that while the 5.5-year-old BIL children in their study differentiated voiceless categories in Spanish and English, they did not differentiate voiced ones; only their 10-year-old BIL children also contrasted /b/ and /d/ in the two languages in line with adult patterns. We would similarly expect that the BIL children in the present study will eventually differentiate voiced stops across Spanish and English. The reason the younger ones did not do so can be explained both by developmental factors and potentially cross-linguistic interactions. With respect to the former, since consistent pre-voicing is articulatorily complex and lacks acoustic salience, substantial input levels are required for fully target-like categories to emerge. It is therefore not surprising that the BIL children produced voiced stops in Spanish with *less pre-voicing* than required for adult-like patterns. At the same time, the children produced English voiced stops with *more pre-voicing* than commonly reported in the literature. This, in turn, may be due to ambiguity in the input, as discussed above, which could have resulted in Spanish and English voiced stop categories being perceptually equated, a phenomenon that is referred to as *equivalence classification* in Flege’s SLM (Flege, 1995; Flege & Bohn, 2021). According to the model, this is more likely to occur where sounds are cross-linguistically similar but not identical, as in the case of voiced stops in English and Spanish.

Our final analysis examined whether age and exposure are predictors for the BIL children’s VOT patterns in the two languages. The results showed that increasing age was associated with lower VOT values for Spanish and English /b/ and /d/, while there was no age effect on voiceless categories in the two languages. Moreover, our binary analysis revealed that pre-voicing is more likely to occur in both languages with increasing age. This finding is in line with Muru and Lee’s (2017) study, which showed that the group of older Spanish-English BIL children was more target-like on voiced stops than the younger BILs, but the two sets of children did not differ on voiceless ones. Age effects are also reported in a number of other studies (McCarthy et al., 2014; Montanari et al., 2023). In contrast, Stoehr et al. (2018) found no effect of age on VOT production in their simultaneous German-Dutch BIL children in the Netherlands. As in the present context, the BIL children in their study were also exposed to an aspirating language and a voicing language. However, unlike the other studies, it was the voicing language, that is Dutch, that constituted the majority language, and hence the children would have benefitted from a “majority language effect” (Gathercole & Thomas, 2009), enhancing experience with the more complex pre-voicing patterns of Dutch at an earlier stage.

Finally, our study found that higher exposure to Spanish was associated with lower VOT values in Spanish and English and a greater probability of pre-voicing in both languages. Children who hear more Spanish were hence more likely to pre-voice their voiced stops, and they were less influenced by the longer VOTs of English in their productions of voiceless stops. These findings largely conform to those of previous studies examining VOT perception and production (Montanari et al., 2023; Ramón-Casas et al., 2023; Stoehr et al., 2018). As in the present study, they found exposure to moderate VOT patterns in the heritage language. Where the present study diverges from them, however, is that it also found greater exposure to the heritage language to affect VOT productions in the majority language. This was somewhat unexpected and suggests CLI patterns from the heritage language to the majority language. Such interactions have been documented in some previous research – Darcy and Krüger (2012), for instance, found that early Turkish-German BIL children in Germany were less accurate than MON German-speaking children in the perception of German vowel contrasts known to be difficult for MON Turkish-speaking adults, which suggests an influence of the children’s heritage language–although they are comparatively rare. Future research will need to build on these findings and examine the specific circumstances in which transfer from the heritage language to the majority language occurs in the speech patterns of BIL children.

## 5. Conclusion

In conclusion, the present study is the first to compare the speech patterns of BIL children to those of closely matched functional MONs from the same community. As such, it provides a socially and culturally more appropriate control setting than in studies where BILs and MONs are assessed in separate communities, often in geographically distant locations. Investigating participants from the same community also has important cognitive consequences in that not only BILs but also MONs are regularly exposed to linguistic items from the community language, which, in turn, may affect speech patterns in the majority language. In the present study, this may have manifested in greater use of pre-voiced tokens in the functional MONs’ English voiced stops than might be expected in MON English settings without language contact. In addition, the present study provides the largest acoustic database of stop productions by Spanish-English BIL children and their MON peers, extending and refining the preliminary findings from previous studies. This is significant in view of the substantial numbers of Spanish speakers in California and the importance that the acquisition of speech sounds plays in early literacy development.

Our study showed that Spanish-English BIL children and matched functional MON children from the same Latinx community differed from each other in their English VOT productions for voiced stops, but not voiceless ones, with lower values exhibited by the BILs. At the same time, the two groups of children did not show a credible difference in the number of pre-voiced tokens for English voiced stops. Nevertheless, they both produced a considerable number of pre-voiced items, which could be a result of CLI with Spanish or input in Spanish-accented English, although additional research is needed to elucidate this matter further. Moreover, the current study showed that Spanish-English BIL 3-to-6-year-olds were able to differentiate voiceless stops across languages, but not voiced ones, and that their VOT productions were significantly affected by age and exposure level.

As with all research, our study has some limitations. First, our measure for exposure was somewhat unsophisticated by merely differentiating children on the basis of parental responses to a single question on a Likert scale ranging from “child hears mostly Spanish” to “child hears mostly English.” While we maintain that this is a time-efficient and reliable measure (Calandruccio et al., 2021), it is possible that a more refined approach, perhaps including absolute numbers, may exhibit even stronger effects and account for a larger amount of the variance. Moreover, a better understanding of the quality of the input patterns that children from Latinx communities receive will make it possible to examine the extent to which functional MONs are indeed solely or primarily affected by hearing Spanish as opposed to Spanish-accented English. Finally, new insights would be gained by future work that tracks the speech productions of Spanish-English BIL children over time and examines their individual variation patterns. Together, such work will bring us a step closer to understanding the complexity involved in the development of speech patterns by children growing up in Latinx communities.

## Supplementary Material

Supplementary Material

## Figures and Tables

**Table 1.** Experimental materials

Target sound	English		Spanish	
	Target word	Transcription	Target word	Transcription with gloss
/p/	*potty*	/'pαɾi/	*panza*	/’pansa/ “belly”
	*puppy*	/'pʌpi/	*papas*	/’papas/ “potatoes”
	*puzzle*	/'pʌz^ə^l/	*pato*	/’pato/ “duck”
/b/	*bottle*	/'bαɾ^ə^l/	*barco*	/’baɾko/ “boat”
	*bubbles*	/'bʌb^ə^lz/	*vaca*	/’baka/ “cow”
	*bunny*	/'bʌni/	*vaso*	/’baso/ “glass”
/t/	*talking*	/tαkɪŋ/	*taco*	/’tako/ “taco”
	*tummy*	/'tʌmi/	*taza*	/’tasa/ “cup”
	*tunnel*	/'tʌn^ə^l/	*torta*	/’toɾta/ “cake”
/d/	*daddy*	/'dædi/	*dama*	/’dama/ “lady”
	*doctor*	/'dαktɚ/	*danza*	/’dansa/ “dance” (n.)
	*doggie*	/'dαɡi/	*dado*	/’daðo/ “die” (n.)

**Table 2.** Mean VOT (in ms), standard deviations (SD) and ranges of the monolingual and bilingual children’s stops

		English				Spanish			
		/p/	/b/	/t/	/d/	/p/	/b/	/t/	/d/
BIL	Mean	63.94	−20.03	74.43	−0.67	17.15	−7.98	19.51	−3.12
	SD	42.64	57.97	38.62	40.86	14.57	45.99	14.96	55.27
	Range	250	282	206	240	94	228	97	358
	Min	−22	−215	4	−177	1	−195	1	−201
	Max	228	67	210	63	95	18	98	157
	*n*	102	105	107	107	104	96	109	103
MON	Mean	58.23	−4.61	73.87	9.61	-	-	-	-
	SD	35.99	45.7	36.86	29.77				
	Range	192	257	218	226				
	Min	3	−209	4	−131				
	Max	195	48	222	95				
	*n*	104	106	104	103				

## Highlights

- Functional monolingual and bilingual children produce English voiceless stops alike
- Bilinguals produce English voiced stops with lower VOT values than monolinguals
- Both monolinguals and bilinguals considerably pre-voice English voiced stops
- Age increases bilinguals’ likelihood of pre-voicing in both Spanish and English
- Greater exposure to English results in less pre-voicing across both languages
