# Supplementary material for: Community language exposure affects voice onset time patterns in Spanish-English bilingual children and functional English monolingual children
Source: Biling (Camb Engl). Author manuscript; Available in PMC 2025 Aug 25. (PMC12373127; doi:10.1017/s1366728925000045)
Supplement: Supplementary Material [file NIHMS2077856-supplement-Supplementary_Material.pdf]

Table S1. *Model summary for the model analysing numerical VOT values in bilingual and monolingual children.*

	**Estimate**	**Err.**	**95%CrI**
**Intercept**	**2.52**	**0.13**	**[2.25, 2.79]**
**place**	**0.65**	**0.19**	**[0.24, 1.05]**
**voicing**	**2.74**	**0.25**	**[2.24, 3.23]**
group	0.40	0.23	[-0.05, 0.85]
place × voicing	-0.60	0.35	[-1.36, 0.15]
place × group	-0.17	0.28	[-0.73, 0.38]
**voicing × group**	**-0.84**	**0.41**	**[-1.66, -0.02]**
place × voicing × group	0.42	0.49	[-0.54, 1.41]

Table S2. *Model summary for the model analysing binary voicing in /b/ and /d/ in bilingual and monolingual children.*

	**Estimate**	**Err.**	**95%CrI**
**Intercept**	**-2.61**	**0.46**	**[-3.61, -1.67]**
place	-0.95	0.63	[-2.21, 0.54]
group	-1.01	0.64	[-2.32, 0.25]
place × group	0.37	0.72	[-1.04, 1.78]

Table S3. *Model summary for the model analysing numerical VOT values in Spanish and English productions from bilingual children. Effects for which 95%CrI exclude the value of 0 are bolded.*

	Estimate	Err.	95%CrI
**Intercept**	**2.00**	**0.15**	**[1.71, 2.30]**
**place**	**0.54**	**0.16**	**[0.21, 0.87]**
**voicing**	**2.38**	**0.26**	**[1.87, 2.89]**
**language**	**-0.62**	**0.19**	**[-0.99, -0.24]**
**age**	**-0.52**	**0.14**	**[-0.81, -0.23]**
**exposure**	**0.33**	**0.15**	**[0.01, 0.63]**
place × voicing	-0.62	0.31	[-1.26, 0.03]
place × language	-0.47	0.33	[-1.15, 0.22]
**voicing × language**	**-1.55**	**0.41**	**[-2.39, -0.71]**
place × age	0.12	0.14	[-0.16, 0.39]
**voicing × age**	**1.10**	**0.25**	**[0.62, 1.60]**
language × age	-0.03	0.17	[-0.37, 0.30]
place × exposure	0.05	0.15	[-0.25, 0.34]
voicing × exposure	-0.39	0.26	[-0.91, 0.15]
language × exposure	0.15	0.18	[-0.21, 0.50]
age × exposure	0.04	0.15	[-0.26, 0.34]
place × voicing × language	0.50	0.60	[-0.71, 1.69]
place × voicing × age	-0.35	0.27	[-0.89, 0.19]
place × language × age	-0.14	0.30	[-0.73, 0.46]
voicing × language × age	-0.34	0.38	[-1.10, 0.43]
place × voicing × exposure	-0.06	0.29	[-0.63, 0.52]
place × language × exposure	-0.25	0.32	[-0.89, 0.37]
voicing × language × exposure	-0.48	0.40	[-1.30, 0.32]
place × age × exposure	0.22	0.15	[-0.07, 0.51]
voicing × age × exposure	-0.12	0.26	[-0.65, 0.40]
language × age × exposure	0.33	0.17	[-0.02, 0.68]
place × voicing × language × age	0.56	0.51	[-0.48, 1.57]
place × voicing × language × exposure	0.31	0.54	[-0.74, 1.38]
place × voicing × age × exposure	-0.26	0.28	[-0.83, 0.30]
place × language × age × exposure	-0.48	0.31	[-1.11, 0.13]
voicing × language × age × exposure	-0.50	0.39	[-1.30, 0.29]
place × voicing × language × age × exposure	0.49	0.54	[-0.60, 1.55]

Table S4. *Model summary for the model analysing binary voicing in /b/ and /d/ in values in Spanish and English productions from bilingual children. Effects for which 95%CrI exclude the value of 0 are bolded.*

	**Est.**	**Err.**	**95%CrI**
**Intercept**	**-2.54**	**0.47**	**[-3.56, -1.68]**
place	-0.73	0.57	[-1.89, 0.48]
language	0.19	0.64	[-1.09, 1.51]
**age**	**1.62**	**0.42**	**[0.80, 2.54]**
**exposure**	**-1.06**	**0.44**	**[-2.00, -0.18]**
place × language	0.63	0.97	[-1.30, 2.47]
place × age	-0.36	0.47	[-1.33, 0.57]
language × age	-0.51	0.54	[-1.61, 0.57]
place × exposure	0.35	0.51	[-0.62, 1.40]
language × exposure	0.09	0.58	[-1.01, 1.32]
age × exposure	0.15	0.41	[-0.69, 1.00]
place × language × age	0.34	0.82	[-1.24, 2.03]
place × language × exposure	0.71	0.87	[-1.02, 2.44]
place × age × exposure	-0.28	0.44	[-1.18, 0.58]
language × age × exposure	-0.79	0.53	[-1.91, 0.26]
place × language × age × exposure	0.28	0.81	[-1.35, 1.92]

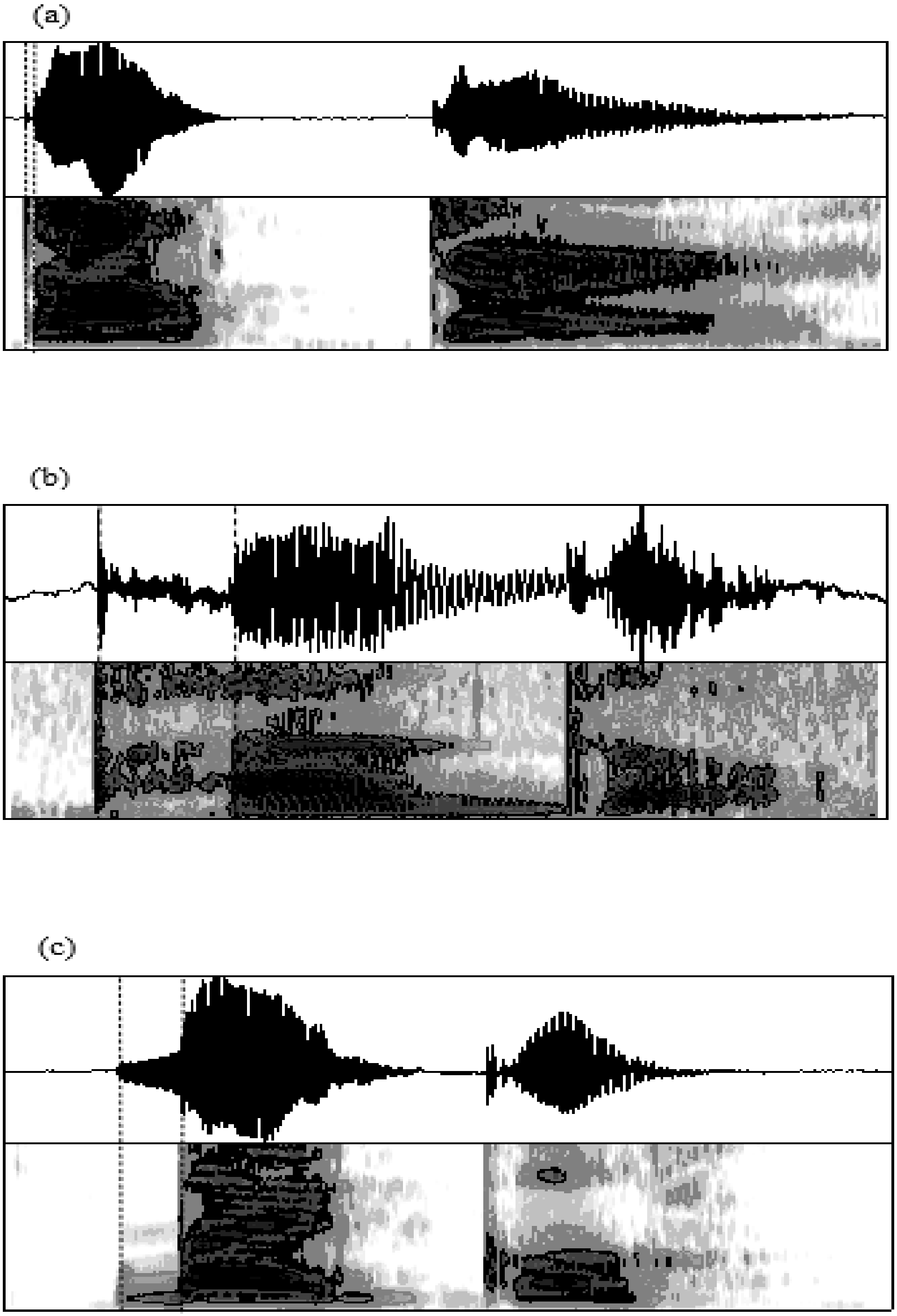

Figure S1. *Waveform and spectrogram of stop realised with short-lag VOT (a), long-lag VOT (b) and lead VOT (c); VOT indicated between broken vertical lines.*
